# The prevalence of dyslipidemia in patients with diabetes mellitus of ayurveda Hospital

**DOI:** 10.1186/2251-6581-13-58

**Published:** 2014-05-22

**Authors:** Amit Kumar Dixit, Ranjit Dey, Aela Suresh, Siddhartha Chaudhuri, Ashok Kumar Panda, Achintya Mitra, Jayram Hazra

**Affiliations:** 1 Department of Pathology and Biochemistry, National Research Institute of Ayurvedic Drug Development, Department of AYUSH, Government of India, 4 CN Block, Sec-5, Bidhannagar, Kolkata 700091, West Bengal, India; 2 Hospital Division, National Research Institute of Ayurvedic Drug Development, Department of AYUSH, Government of India, 4 CN Block, Sec-5, Bidhannagar, Kolkata 700091, West Bengal, India

**Keywords:** Diabetes mellitus, Blood sugar, Dyslipidemia, Hyperlipidemia, Lipid profile

## Abstract

**Background:**

Dyslipidemia is one of the major risk factors for cardiovascular disease in diabetes mellitus. Early detection and treatment of dyslipidemia in type-2 diabetes mellitus can prevent risk for atherogenic cardiovascular disorder. The rationale of this study was to detect the lipid abnormality in diabetic patients.

**Methods:**

Necessary data was collected from the medical archives of 150 patients (73 female and 77 male) with diabetes mellitus registered in Department of pathology and biochemistry of a Ayurveda hospital established at Kolkata, India.

**Results:**

The mean ages of female and male subjects were 51.8 ± 10.8 and 53.2 ± 11.3 years respectively. The range and mean value of FBS in females were 113–342 mg/dl and 157.7 ± 6.3 mg/dl, while the range and mean value of PPBS in females were 135–560 mg/dl and 275.5 ± 12.3 mg/dl respectively. Results showed that range and mean value of FBS in males were 111–462 mg/dl and 160.8 ± 7.4 mg/dl, while the range and mean value of PPBS in males were 136–598 mg/dl and 302.1 ± 12.6 mg/dl respectively. Results of serum lipids showed that the mean values for total cholesterol (TC), triglyceride (TG), high density lipoprotein cholesterol (HDL-C), low density lipoprotein cholesterol (LDL-C) and very low density lipoprotein cholesterol (VLDL-C) in female patients were 202.2 ± 5.9 mg/dl, 168.3 ± 8.2 mg/dl, 44.9 ± 1.3 mg/dl, 123.6 ± 5.2 mg/dl and 33.7 ± 1.7 mg/dl respectively. The mean values for TC, TG, HDL-C, LDL-C and VLDL-C in male patients were 182.5 ± 4.8 mg/dl, 128.1 ± 10.8 mg/dl, 40.8 ± 1.2 mg/dl, 105.4 ± 4.8 mg/dl and 36.2 ± 2.2 respectively. FBS showed significant positive correlation with PPBS, cholesterol, TG, and VLDL-C. PPBS also demonstrated direct and significant correlations with TG and VLDL-C.

**Conclusions:**

The study showed common lipid abnormalities during diabetes induced dyslipidemia i.e., hypercholesterolemia, hypertriglyceridemia and elevated LDL-C. This study suggests the dominance of hyperlipidemia over increased prevalence of dyslipidemia.

## Background

The frequency of diabetes mellitus is increasing many folds in South Asian population due to the high degree of genetic predisposition and high susceptibility to environmental insulin, characterized by a high BMI, high upper body adiposity, a high body fat percentage and a high level of insulin resistance [1]. All forms of diabetes are characterized by absolute or relative deficiencies in insulin secretion and/or insulin action associated with chronic hyperglycemia and disturbances of carbohydrate, lipid and protein metabolism [2]. The chronic hyperglycemia of diabetes is associated with long-term damage, dysfunction, and failure of various organs, especially the eyes, kidneys, nerves, heart, and blood vessels [3].

Research finding shows that it is the body composition components, mainly body fat and lipid profiles that are responsible for increase prevalence of this disease [2,4]. he term “dyslipidemia” is increasingly popular to describe abnormal changes in lipid profile, replacing the old term “hyperlipidemia”. Dyslipidemia encompasses changes in High density lipoprotein cholesterol (HDL-C), the size and density of Low density lipoprotein cholesterol (LDL-C), very low density lipoprotein cholesterol (VLDL-C) and triglyceride level [5-7]. The term diabetic dyslipidemia comprises a triad of raised triglycerides, reduced HDLC and excess of small, dense LDL particles. The lipid abnormalities are prevalent in diabetes mellitus because insulin resistance or deficiency affects key enzymes and pathways in lipid metabolism [8]. In particular, the following processes are affected: apoprotein production, regulation of lipoprotein lipase, action of cholesteryl ester, transfer proteins and hepatic and peripheral actions of insulin [4,5]. Even more, it has been proposed that the composition of lipid particles in diabetic dyslipidemia is more atherogenic than other types of dyslipidemia [9]. This means that even normal lipid concentrations might be more atherogenic in diabetic than in nondiabetic people. The causal association between atherosclerosis and dyslipidemia is well established. In diabetes the associated hyperglycemia, obesity and insulin changes highly accelerate the progression to atherosclerosis [10,11].

Therefore, present study was carried out to find association/relation between serum lipid profile and blood sugar, in view of the hypothesis that early detection and treatment of lipid abnormalities can minimize the risk for atherogenic cardiovascular disorder and cerebrovascular accident in type-2 diabetic patients. Hence, the rationale of this study was to detect dyslipidemia in patients with type-2 diabetes mellitus.

## Material and methods

### Chemical

The diagnostic kits i.e. Glucose, Cholesterol, triglyceride, HDL-C, LDL-C VLDL-C and reagents were procured from Siemens Ltd, (Gujrat India). All the reagents were stored at 2-8ºC after procurement. All the biochemical estimations were performed at room temperature.

### Subjects

It is a retrospective study based on the available biochemical data of type-2 diabetic patients visiting the Biochemistry department for the diagnosis of blood sugars and lipid profile, after prescription and medication from OPD and IPD of the Ayurveda hospital at Kolkata, India. A total of 150 diabetic patients (73 females and 77 males) with age range of 36–75 years and with history of diabetes for more than 4 years were randomly selected from the medical records. Patients reported with other ailments and metabolic disorders were excluded from the study. The individual information about clinical symptoms, weight, height and diagnosis by the hospital physicians were well documented in medical records of the hospital.

### Estimation of blood sugar

Venous blood samples were collected from all the subjects in the morning after fasting overnight. The plasma was later used for analyzing FBS and PPBS. Plasma glucose was measured by Glucose oxidase and peroxidase (GOD-POD) method. It is based on the principle that glucose is oxidized by glucose oxidase into gluconic acid and hydrogen peroxide. Hydrogen peroxide in presence of peroxidase oxidizes the chromogen 4-aminoantiyrine/phenolic compound to a red colored compound. The intensity of the red colored compound is proportional to the glucose concentration and is measured at 505 nm using a semi-automated enzymatic analyzer (Robonik, Mumbai, India).

### Estimation of total cholesterol

Cholesterol esters will be hydrolyzed by cholesterol esterase. Cholesterol will be oxidized into cholest-4-en-3-on and hydrogen peroxide by bacterial cholesterol oxidase. Hydrogen peroxide in the presence of phenol and amino-4-antipyrine forms a complex of red color showing absorption maximum between 500–550 nm.

Estimation of triglyceride: It is based on the principle that triglycerides in the serum sample are hydrolyzed enzymatically by the action of lipase to glycerol and fatty acids. The glycerol formed is converted to glycerol phosphate by glycerol kinase (GK). Glycerol phosphate is then oxidized to dihydroxyacetone phosphate by glycerol phosphate oxidase (GPO). The librated hydrogen peroxide is detected by a chromogenic acceptor, chlorophenol-4-aminoantipyrine, in the presence of peroxidase (POD). The red quinone formed is proportional to the amount of triglycerides present in the sample and is measured at 546 nm.

Estimation of total HDL-cholesterol: It is measured by using phosphotungstate precipitation method based on the principle that chylomicrons, VLDL and LDL fractions in serum or plasma are separated from HDL by precipitating with phosphotungstic acid and magnesium chloride. After centrifugation the cholesterol in the HDL fraction which remains in the supernatant is assayed with enzymatic cholesterol method using cholesterol esterase, cholesterol oxidase, peroxidase and the chromogen 4-aminoantipyrine.

Estimation of total LDL-cholesterol: The LDL-Cholesterol test is a two reagent homogenous system. The assay is comprised of two distinct phases. In phase one a unique detergent solubilizes cholesterol from non-LDL-lipoprotein particles. This cholesterol is consumed by cholesterol esterase, cholesterol oxidase, peroxidase and 4-aminoantipyrine to generate a colorless end product. In phase two a second detergent in reagent 2 releases cholesterol from the LDL–lipoproteins. This cholesterol reacts with cholesterol esterase, cholesterol oxidase and a chromogen system to yield a blue color complex which can be measured bichromatically at 540/660 nm. The resulting increase in absorbance is directly proportional to the LDL-C concentration in the sample.

Estimation of total VLDL-cholesterol: The value of VLDL-cholesterol was calculated as one-fifth of the concentration of triglycerides.

### Statistical analysis

The data obtained was analyzed for significance between the groups by one-way analysis of variance (ANOVA). Correlation studies (Pearson’s correlation coefficients and significance) were performed between the variables of blood sugar and serum lipid profile by using statistical software programme “SPSS evaluation version 22”.

## Results

Results of the retrospective study showed that among 150 patients of diabetes mellitus included in this study, female and male were 73 and 77 respectively (Table 1). The age range and mean age of female subjects was 40–75 years and 51.8 ± 10.8 while the age range and mean age for male subjects was 36–72 years and 53.2 ± 11.3 years, respectively.

**Table 1:** Gender distribution and age of selected type-2 diabetes patients

**Gender**	**No. of patients**	**Age**^**1**^
Female	73	51.8 ± 10.8
Male	77	53.2 ± 11.3
Total	150	52.5 ± 11.1

^1^Units expressed as years.

Results of the blood sugar profile showed that all individuals were hyperglycaemic. The range and mean value of FBS in females were 113–342 mg/dl and 157.7 ± 6.3 mg/dl, while the range and mean value of PPBS in females were 135–560 mg/dl and 275.5 ± 12.3 mg/dl respectively. Results showed that range and mean value of FBS in males were 111–462 mg/dl and 160.8 ± 7.4 mg/dl, while the range and mean value of PPBS in males were 136–598 mg/dl and 302.1 ± 12.6 mg/dl respectively (Table 2). It was observed that some patients have controlled FBS, but there PPBS was uncontrolled. Therefore, the range of FBS in some patients looks nearly normal or controlled, while in few patients it was uncontrolled. Result also showed that PPBS were slightly higher in males as comparison to female madhumeha patients.

**Table 2:** The concentration of fasting blood sugar (FBS) and post prandial blood sugar (PPBS) in plasma of diabetic patients

**Gender**	**Glucose (FBS)**^**1**^			**Glucose (PPBS)**^**1**^		
	**Normal range**	**Calculated range**	**Mean**	**Normal range**	**Calculated range**	**Mean**
Female	70-110	113-342	157.7 ± 6.3*	110-140	135-560	275.5 ± 12.3*
Male	70-140	111-462	160.8 ± 7.4*	70-140	136-598	302.1 ± 12.6*

^1^Units expressed as mg/dl.

*Statistically significant (P < 0.05) as compared to normal range for plasma blood sugar.

Results of serum lipid profile showed that the means values for TC, TG, HDL-C, LDL-C and VLDL-C in female patients were 202.2 ± 5.9 mg/dl, 168.3 ± 8.2 mg/dl, 44.9 ± 1.3 mg/dl, 123.6 ± 5.2 mg/dl and 33.7 ± 1.7 mg/dl respectively. The means values for TC, TG, HDL-C, LDL-C and VLDL-C in male patients were 182.5 ± 4.8 mg/dl, 128.1 ± 10.8 mg/dl, 40.8 ± 1.2 mg/dl, 105.4 ± 4.8 mg/dl and 36.2 ± 2.2 respectively. Results showed that diabetic females have significantly higher (P < 0.05) TC, TG and LDL-C as compared to male patients (Table 3). Among all patients hypercholesterolemia was found in 79 (52.6%) individuals. Similarly, hypertriglyceridemia was found in 95 (63.3%) individuals, and increased LDL-C was found in 108 (72.6%) individuals.

**Table 3:** The concentration of cholesterol, triglyceride, HDL-Cholesterol, LDL-Cholesterol and VLDL-Cholesterol in serum of diabetic patients

**Lipid parameter**	**Female**			**Male**		
	**Normal range**	**Calculated range**	**Mean**	**Normal range**	**Calculated range**	**Mean**
Total Cholesterol^1^	< 200	130-396	202.2 ± 5.9*	< 200	100-295	182.5 ± 4.8*
Triglyceride^1^	50-150	60-380	168.3 ± 8.2*	50-150	60-650	128.1 ± 10.8*
HDL-Cholesterol^1^	45-65	20-66	44.9 ± 1.3	35–55	22-60	40.8 ± 1.2
LDL-Cholesterol^1^	< 100	42-274	123.6 ± 5.2*	< 100	26-237	105.4 ± 4.8*
VLDL-Cholesterol^1^	15-30	12-76	33.7 ± 1.7	15-30	12-130	36.2 ± 2.2

^1^Units expressed as mg/dl.

*Statistically significant (*P* < 0.01) as compared to normal range for respective serum lipids.

In correlation studies, FBS showed significant positive correlation with PPBS (P < 0.001), cholesterol (P < 0.05), TG (P < 0.01) and VLDL-C (P < 0.001) (Table 4). PPBS also demonstrated direct and significant correlations with TG (P < 0.01) and VLDL-C (P < 0.01). The correlation of cholesterol with TG (P < 0.001), LDL-C (P < 0.001) and VLDL-C (P < 0.001) were positive. TG showed a significant negative correlation with HDL-C (P < 0.01), while a highly significant positive correlation was observed with VLDL-C (P < 0.001). However, HDL-C showed a negative correlation with LDL-C and VLDL-C (P < 0.01).

**Table 4:** Correlation studies between the blood sugar and serum lipid profile variables of diabetic patients

	**PPBS**	**Cholesterol**	**TG**	**HDL-C**	**LDL-C**	**VLDL-C**
FBS	0.633***	0.167*	0.292**	-0.021	0.072	0.292***
PPBS		0.063	0.249**	0.108	-0.051	0.249**
Cholesterol			0.317***	0.103	0.931***	0.317***
TG				-.235**	0.009	1.000***
HDL-C					-0.012	-0.235**
LDL-C						0.009

The values expressed as Pearson correlation coefficients.

*Correlation is significant at the 0.05 level.

**Correlation is significant at the 0.01 level.

***Correlation is significant at the 0.001 level.

## Discussion

The relation between diabetes mellitus and serum lipid profile had been much discussed during the past decades [4,5,12,13]. Both lipid profile and diabetes have been shown to be the important predictors for metabolic disturbances including dyslipidaemia, hypertension, cardiovascular diseases, hyperinsulinemia, etc. [6]. Dyslipidemia as a metabolic abnormality is frequently associated with diabetes mellitus. Its prevalence is variable, depending on the type and severity of diabetes, glycaemic control, nutritional status, age and other factors. Earlier studies also indicated a strong clustering risk factor for coronary artery disease in diabetic subjects [4,11]. Over 70% of patients with type 2 diabetes mellitus had one or more types of dyslipidemia. Similarly, our results reveal high prevalence of hypercholesterolemia, hypertriglyceridemia and high LDL-C levels, which are well known risk factors for cardiovascular diseases among patients. These patients are on high-risk without complications but already had significant dyslipidemia, which enhances the risk of cardiovascular events, certainly required therapeutic intervention.

In diabetes many factors may affect blood lipid levels, because of interrelationship between carbohydrates and lipid metabolism. Therefore, any disorder in carbohydrate metabolism leads to disorder in lipid metabolism and vice versa [14]. Insulin resistance is a primary defects in the majority of with type-2 diabetes. In non diabetic individuals insulin resistance in combination with hyperinsulinemia has a strong predictive value for future development for type-2 diabetes [15]. Several studies showed that insulin affects the liver apolipoprotein production and regulates the enzymatic activity of lipoprotein lipase and cholesterol ester transport protein, which causes dyslipidemia in diabetes mellitus. Moreover, insulin deficiency reduces the activity of hepatic lipase and several steps in the production of biologically active lipoprotein lipase [4,5,7].

The result of the present study indicates that the most common recognized abnormality was hypertriglyceridaemia. Other researchers also associated the high triglyceride level to the poor glycaemic control of diabetes and obesity [5,16]. This hypothesis is supported by the reduction of the triglyceride level with the improvement of glycaemic control i.e. FBS and PPBS. Abbate and Brunzell reported that the increase in triglycerides in poorly controlled patients was related to the decrease of activities of adipose tissue and muscle lipoprotein lipase activity [17].

A study by Packard et al., reported that reduced HDL-C as a powerful predicator for premature coronary heart diseases [18]. Goldberg reported that hyperglycemia progressively increases the transfer of cholesterol esters from HDL-C to VLDL-C particles, hence, denser LDL particles acquire a large proportion of these HDL esters, further diminishing the HDL-C level [6]. In addition, HDL-C is a ready substrate for hepatic lipase which converts it into smaller particles, which are readily cleared from the plasma [4,5]. As with the triglycerides, improvement in glycaemic control leads to an increase in the levels of HDL-C, and suggest the evidence for a role for poor glycaemia in decreasing the level of this lipoproteins. Poor insulinization results in increased lipolysis in adipocytes. The resulting increase in fatty acid transport to the liver, which is a common abnormality in type 2 diabetes, may cause an increase in VLDL-C. Insulin directly degrades the apo B (which is the major protein of VLDL particles) and thus insulin may increase secretion of apo B (and then VLDL) [19]. In correlation studies, our results were compatible with the finding of Rosediani et al., who reported significant positive correlation of FBS with PPBS [20]. Results of our correlation studies demonstrated significant positive correlation of blood sugar with, cholesterol, TG and VLDL-C. Similarly, cholesterol showed positive correlation with TG, LDL-C and VLDL-C. Significant negative correlation of TG with HDL-C in the present study was in agreement with the earlier study of Mahato et al. [9]. However, HDL-C showed a negative correlation with LDL-C and VLDL-C which. Results of the correlation studies suggest a clear association between hyperglycemia and appearance of dyslipidemia. Similarly, Tseng has also reported lipid abnormalities in patients with type 2 diabetes with regards to different stages of albuminuria and suggested a close link between uric acid and increased urinary albumin excretion rate in type 2 diabetic patients of Taiwan [21,22]. Several authors reported a positive improvement in lipid profile with fair glycaemic control [3,23]. In uncontrolled diabetic patients, it has been reported that the activity of lipoprotein lipase and hence clearance of VLDL-C in the circulation is diminished due to insulin resistance. The level of total cholesterol is usually normal or near normal if glycaemic control is adequate, and worsening of control raises the level [24]. Therefore, improving glycaemic control can substantially reduce the risk of cardiovascular events in diabetic patients. As the occurrence of dyslipidemia depends on factors such as insulin action on peripheral tissues and liver, apoprotein production and regulation of lipoprotein lipase, the duration of diabetes seems to play only a minor role in modifying these factors [14]. In addition, clinicians tend to neglect looking for other secondary causes of dyslipidemia, such as renal disease, alcohol consumption and certain drugs. These factors may accelerate the progression of dyslipidemia at any stage of diabetes (i.e. irrespective of duration).

Dyslipidemia management in people with diabetes mellitus, just like in any other individual, starts with a thorough evaluation that aims to identify secondary causes that might contribute to the abnormal lipid profile [25]. Lifestyle changes, including increased physical activity and dietary modifications, are the cornerstones of management. The highest priority for diabetic individuals who have poor glycaemic control should be to achieve near normal blood glucose levels, in the expectation that this approach will also improve dyslipidemia [5,26,27]. Our study clearly shows that lipid fractions are abnormal in diabetes mellitus. Realizing that most of the diabetics have a high probability of developing cardiovascular and cerebrovascular disease, it is essential that an individual who is diabetic should take care of dyslipidemia. Our study clearly suggests the dominance of hyperlipidemia over increased prevalence of dyslipidemia. Prospective and longitudinal studies are needed at the time of diagnose to estimate true population prevalence of dyslipidemia in patients with type-2 diabetes mellitus.

## Conclusions

Present study showed common lipid abnormalities during diabetes induced dyslipidemia are hypercholesterolemia, hypertriglyceridemia and elevated LDL-C. Results suggest a high prevalence of dyslipidemia, which might be playing a major role in the development of cardiovascular diseases among diabetic patients. The optimal care of diabetic patients should include routine monitoring of blood sugar and serum lipid profile. Aggressive lifestyle changes, such as weight reduction and physical exercise should be initiated first followed by medication with lipid lowering drugs. The optimum treatment with antidiabetic drugs to obtain fair glycaemic control should go hand-in-hand with lipid-lowering drugs.

## Competing interests

The authors declared that they have no competing interests.

## Authors’ contribution

AKD contributed to conception, designation of the study, acquisition, analysis of data and drafted the manuscript. RK, AS, SC participated in study designed and analysis. AKP and AM participated in study design. JH helped in coordination and gave final approval of the version to be published. All authors read and approved the final manuscript.
